# Safety of Same-Day Discharge After Elective Transcatheter Aortic Valve Implantation With Balloon- and Self-Expanding Valves: A Prospective Single-Center UK Study

**DOI:** 10.1016/j.shj.2025.100728

**Published:** 2025-09-05

**Authors:** Saif Memon, Muntaser Omari, Debbie Stewart, Hong Hong Chong, Mohamed Ali, Richard Edwards, Rajiv Das, Tim Cartlidge, Azfar Zaman, Mohamed Farag, Mohammad Alkhalil

**Affiliations:** a Cardiothoracic Centre, Freeman Hospital, Newcastle-upon-Tyne, UK; b Translational and Clinical Research Institute, Newcastle University, Newcastle-upon-Tyne, UK

**Keywords:** Balloon expandable, Outcome, Same-day discharge, Self-expanding, TAVI

## Abstract

**Background:**

In patients undergoing transcatheter aortic valve implantation (TAVI), it is unclear whether same-day discharge (SDD) while adopting a device-agnostic approach is a safe strategy compared to non-SDD. We aim to compare TAVI patients who underwent SDD and non-SDD according to a predefined protocol.

**Methods:**

This is a prospective single-center study of consecutive patients who were scheduled for elective TAVI procedures. The primary endpoint was the composite of death, vascular access–related complications, any bleeding requiring hospitalization, all stroke (or transient ischemic attack), or new permanent pacemaker implantation at 30 days defined according to the Valve Academic Research Consortium 3 criteria.

**Results:**

Out of 472 consecutive patients who underwent elective TAVI, 289 patients did not report procedural complications, of whom 60 (21%) were discharged on the same day. The mean age was 81 ± 7 years, with 60% of the cohort being male. There were no significant differences in clinical characteristics among patients according to their in-hospital SDD strategy. Pre-existing left or right bundle branch block was comparable between the 2 groups. The primary endpoint was reported in 2.8% of the entire cohort, with no significant difference between patients who underwent SDD TAVI and those who did not (1.7% vs. 3.1%, respectively, *p* = 0.56). Patients who had a self-expanding valve had a comparable primary endpoint to those who had a balloon-expandable valve, including readmission for a permanent pacemaker.

**Conclusions:**

SDD TAVI is a safe and feasible approach in patients who underwent an elective uneventful procedure, including patients who received self-expanding valves. Future studies are required to support these findings.

## Highlights

• Same-day discharge following elective trans-catheter aortic valve implantation appears safe for a selected group of patients.
• Same-day discharge was not associated with increased risk of 30-day pacemakers.

## Graphical abstract

Summary of clinical outcomes of patients undergoing same-day versus non–same-day discharge: Patients who met inclusion criteria for same-day discharge were compared with those who stayed in hospital for at least one night, and outcomes, including death, vascular complications, neurological events, and late pacemaker, were assessed at 30 days.

**Figure:**
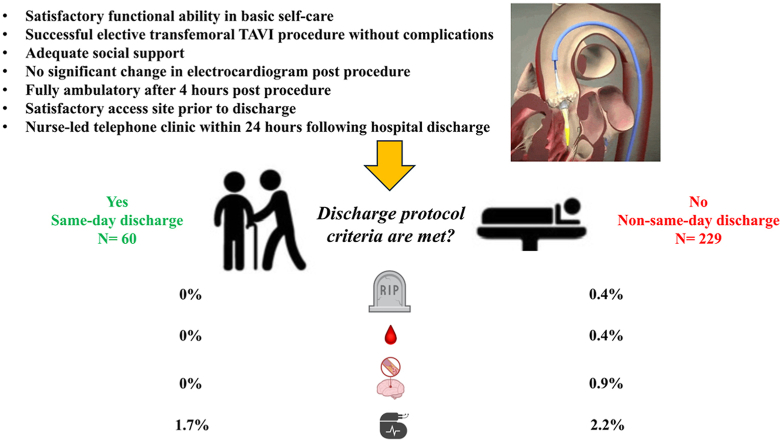

## Introduction

Transcatheter aortic valve implantation (TAVI) is standard treatment for patients with symptomatic severe aortic stenosis who are at different surgical risks.^1^^,^^2^ More recently, patients with low surgical risk had comparable outcomes to surgical aortic valve replacement (AVR) at 5-year follow-up.^3^^,^^4^ All studies showed TAVI to consistently result in shorter length of stay and early hospital discharge compared to surgical AVR.^5^^,^^6^ It has also been associated with higher likelihood of patients being discharged home, as opposed to the rehabilitation facilities following surgical AVR, with better functional status, improved quality of life, and reduced burden on healthcare providers.^5^^,^^7^

The safety of next-day discharge following TAVI has been documented using a standardized and optimized clinical pathway to ensure patient safety.^8^^,^^9^ More recently, the safety of same-day discharge (SDD) following TAVI has also been reported.^10^^,^^11^ This was mainly driven during COVID-19 pandemic to preserve essential hospital resources, and the reported studies included highly selected patients not reflective of real-world practice.^10^^,^^11^ In fact, some studies reported higher incidence of 30-day readmission in patients with same-day compared to next-day discharge.^12^

Advances in procedural techniques, valve technology, and institutional and operator experience have significantly improved procedural safety and outcomes. The use of minimalist approaches allowed the concept of SDD to become a possibility, leading to efficient utilization of hospital resources, early mobilization, increased patient satisfaction, and decreased exposure to hospital-acquired infections. This study reports the clinical characteristics and outcomes of patients who underwent SDD TAVI in a large center following implementation of a predefined protocol to identify patients suitable for SDD.

## Methods

### Study Design

This was a prospective analysis of consecutive patients who underwent elective TAVI procedure. Data were entered into a dedicated database that was reviewed and maintained by a nurse specialist and regularly monitored and audited to ensure data accuracy and completeness. Clinical, procedural, and echocardiographic data were collected from this database for analysis.

### Study Population

Between January 2024 and January 2025, consecutive patients with severe native aortic stenosis or degenerative surgical bio-prosthesis valve who were scheduled for elective TAVI procedure were included. Patients who were transferred for in-hospital TAVI or those who developed procedural complications, including the need for a permanent pacemaker (PPM), were excluded from the analysis. All patients were discussed at and approved for TAVI by the local heart team in accordance with current guidelines.^1^ Patients were included irrespective of surgical risk, planned valve platform, presence of left ventricular (LV) dysfunction, or low flow state. All patients underwent multidetector computed tomography as part of their procedural planning, and images were analyzed using 3mensio software (3mensio Structural Heart, 3mensio Medical Imaging, Maastricht, The Netherlands), as previously described.^13^^,^^14^

TAVI procedures were performed by 2 experienced operators. The decision to choose a particular TAVI device, perform balloon valvuloplasty, postdilatation, and device closure was left to the operator's discretion. TAVI devices included both self-expanding platforms (Evolut [Medtronic, Minneapolis, Minnesota, USA], Navitor [Abbott, Illinois, USA], Allegra [Biosensors International, Morges, Switzerland], and Hydra [SMT, Surat, Gujarat, India]) and balloon-expandable platforms (SAPIEN [Edwards Lifesciences, 10.13039/100008476Irvine, California, USA] and Octacor [Meril Lifesciences, Vapi, Gujarat, India]).

#### Same-day discharge protocol

All elective TAVI patients over the study period were considered for SDD if they fulfilled predefined criteria (Supplementary Table).

Patients attended a nurse-led clinic assessment 1 week prior to their TAVI procedure to have the procedure explained, obtain consent forms and baseline bloods, and inform patients about suitability for SDD. Patients were subsequently admitted on the day of TAVI procedure and scheduled in the morning session. However, patients who were scheduled for early afternoon sessions were also considered if they had at least 6 hours of postprocedural monitoring.

Patients needed to fulfil predefined criteria to be discharged on the same day following their TAVI procedure. These include:

1. Satisfactory patient functional ability in basic self-care (defined as Katz score above 4), including cognitive function.
2. Successful transfemoral TAVI procedure without vascular or neurological complications or significant paravalvular regurgitation.
3. Adequate social support, including the availability of an individual to stay with the patient for the first night.
4. No significant change in electrocardiogram immediately after the procedure and before discharge for patients without cardiac devices with no significant conduction abnormalities on 4 hours of telemetry.
5. Contrast volume less than 3 times the estimated glomerular filtration rate.
6. Fully ambulatory 4 hours after the procedure (supine for 2 hours and sitting up for 1 hour following the procedure).
7. Satisfactory access site with no significant bleeding or hematoma prior to discharge.
8. The availability of TAVI Specialist Nurse to contact patients within 24 hours following hospital discharge.

Patients with pre-existing bundle branch block (BBB) who did not develop significant changes following TAVI were considered for SDD, if they met the rest of the inclusion criteria. Those who developed left bundle branch block (LBBB) immediately after the procedure or at any time during their in-hospital stay were labelled as new-onset LBBB. Both transient and persistent LBBB were included in this group.

#### Study Endpoints

The primary endpoint was any one or more of the following: death, vascular access-related, complications, any bleeding requiring hospitalization, all stroke (or transient ischemic attack), or new PPM implantation at 30 days. All endpoints were defined according to the Valve Academic Research Consortium 3.^15^ The secondary endpoints included the individual components of the primary endpoint at 30 days following hospital discharge.

Patients who died or developed in-hospital complications, access-site vascular complications, PPM, or stroke were excluded from this study.

The study was part of a clinical audit, and formal research ethics approval was not required. All patients provided written informed consent for their procedures, and the study was conducted according to the Declaration of Helsinki and Good Clinical Practice guidelines.

### Statistical Analysis

Data were tested for normality using Shapiro-Wilk test. Normally distributed data were assessed using unpaired *t* test (presented as mean and standard deviation), while non-normally distributed variables were compared using Wilcoxon rank sum test (presented as median and interquartile range) between the 2 groups, namely patients who underwent SDD versus non-SDD. Categorical variables were compared using χ^2^ test or Fisher's exact test, as appropriate. Kaplan-Meier curves were constructed, and the associated log-rank test was calculated to determine the differences in the primary endpoint according to the patient SDD strategy. A multivariate logistic regression analysis was conducted to adjust for the imbalance between the 2 groups, including variables that had a *p* value <0.1. A *p* value <0.05 was considered significant. All statistical analyses were performed using SPSS 29.0 (SPSS, Inc, Chicago, Illinois).

## Results

**Figure 1:**
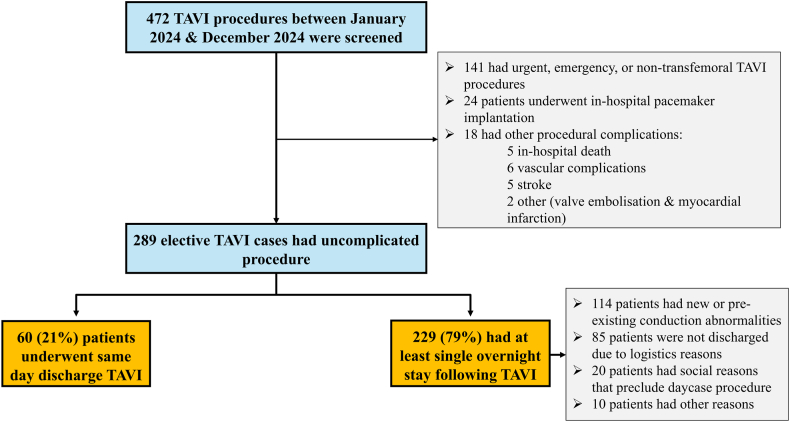
Study flowchart. Abbreviation: TAVI, trans-catheter aortic valve implantation.

Over the study period, 472 patients underwent TAVI procedures at the Freeman Hospital. Patients who did not meet study inclusion criteria, including those who underwent urgent or emergency TAVI, nontransfemoral approach, or develop in-hospital complications were excluded from the analysis (Figure 1). Out of 472 patients, 141 (30%) received TAVI following in-hospital admission (urgent or emergency) or via nontransfemoral approach. Of the remaining 331 elective transfemoral TAVIs, 24 (7.3%) patients underwent pacemaker for persistent high-degree atrioventricular block (AV block), and 18 (5.4%) patients developed procedural complications, including in-hospital death (5 patients), vascular complications (6 patients), stroke (5 patients), valve embolization (1 patient), and myocardial infarction (1 patient).

There were 289 patients who underwent uncomplicated elective TAVI. Of those, 60 patients (21%) were discharged on the same day while the remaining 229 (79%) had at least one night in hospital. New or pre-existing conduction abnormalities in 114 patients (50%) (such as LBBB or first-degree AV block), logistics (37%), and social reasons (9%) were the main reasons for keeping patients in hospital overnight (Figure 1). There were 10 patients who were kept in hospital as they had unsatisfactory access-site (that did not require invasive intervention) or reported nonspecific symptoms, and the responsible physician decided to keep them in hospital for additional monitoring.

The mean age was 81 ± 7 years, with 60% of the cohort being male. The mean gradient was 46 ± 12 in the whole cohort, and 92% had preserved LV function (ejection fraction of more than 50%) on transthoracic echocardiogram before TAVI. There were no significant differences in age, clinical and echocardiographic characteristics among patients according to their in-hospital SDD strategy, except for baseline creatinine level, which was lower in patients undergoing SDD TAVI than in those who had at least one night in-hospital stay (87 ± 29 vs. 98 ± 42, *p* = 0.02) (Table 1).

**Table 1:** Baseline clinical and echocardiographic characteristics of included patients

Baseline characteristic	Whole cohort (*n* = 289)	Non–same-day discharge TAVI (*n* = 229)	Same-day discharge TAVI (*n* = 60)	*p* Value
Age, y	81 ± 7	82 ± 7	80 ± 5	0.065
Gender, male gender	172 (60%)	137 (60%)	35 (58%)	0.83
Body mass index, kg/m^2^	29 ± 6	28 ± 7	29 ± 5	0.25
STS score	2.5 ± 1.8	2.7 ± 1.9	2.0 ± 0.9	<0.001
Diabetes	63 (22%)	53 (23%)	10 (17%)	0.28
Previous smoker	111 (38%)	86 (38%)	25 (42%)	0.56
Obstructive lung disease	11 (4%)	11 (5%)	0 (0%)	0.083
Creatinine, mmol/L	96 ± 40	98 ± 42	87 ± 29	0.022
Chronic kidney disease	103 (36%)	86 (38%)	17 (28%)	0.18
Previous CVA/TIA	15 (5%)	14 (6%)	1 (2%)	0.17
Atrial fibrillation	65 (23%)	54 (24%)	11 (18%)	0.39
NYHA III/IV	177 (61%)	142 (62%)	35 (58%)	0.60
Pre-existing RBBB	16 (6%)	13 (6%)	3 (5%)	0.84
Pre-existing LBBB	33 (11%)	27 (12%)	6 (10%)	0.70
Pacemaker in situ	18 (6%)	10 (4%)	8 (13%)	0.011
Mean gradient, mmHg	46 ± 12	46 ± 13	45 ± 11	0.59
Peak gradient, mmHg	74 ± 21	75 ± 22	71 ± 17	0.29
Aortic valve area, cm^2^	0.75 ± 0.32	0.75 ± 0.35	0.75 ± 0.18	0.95
Degenerative aortic valve	280 (97%)	223 (97%)	57 (95%)	0.35
Moderate MR	4 (1%)	4 (2%)	0 (0%)	0.30
Ejection fraction	54 ± 5	54 ± 5	55 ± 1	<0.001
Preserved LV function	266 (92%)	207 (90%)	59 (98%)	0.043

*Note.* Baseline transthoracic echocardiography parameters are reported within 4 mo of the TAVI procedure. Data presented as mean ± standard devation or n (%), where applicable.

Abbreviations: CVA, cerebrovascular event; LBBB, left bundle branch block; LV, left ventricle; MR, mitral regurgitation; NYHA, New York Heart Association; RBBB, right bundle branch block; STS, Society of Thoracic Surgeons; TAVI, transcatheter aortic valve implantation; TIA, transient ischemia attack.

The proportion of patients who had pre-existing LBBB (10% vs. 12%, respectively, *p* = 0.70) or right bundle branch block (RBBB; 5% vs. 6%, respectively, *p* = 0.84) was comparable between SDD versus non-SDD patients. However, there was higher percentage of patients who had previous pacemaker in the SDD TAVI group (13% vs. 4%, *p* = 0.011).

There was no difference in the proportion of patients who underwent self-expanding valve among patients who did or did not undergo SDD TAVI (52% vs. 59%, respectively, *p* = 0.31). Following TAVI, LBBB was less frequently encountered in the SDD TAVI group (23% vs. 44%, *p* = 0.03), although the duration of QRS complex was comparable between the 2 groups (12 [0-48] vs. 9 [4-19] milliseconds, *p* = 0.19). Similarly, there was no significant difference in the change of PR interval following TAVI between the 2 groups (13 ± 57 vs. 15 ± 26 milliseconds, *p* = 0.68) (Table 1).

**Figure 2:**
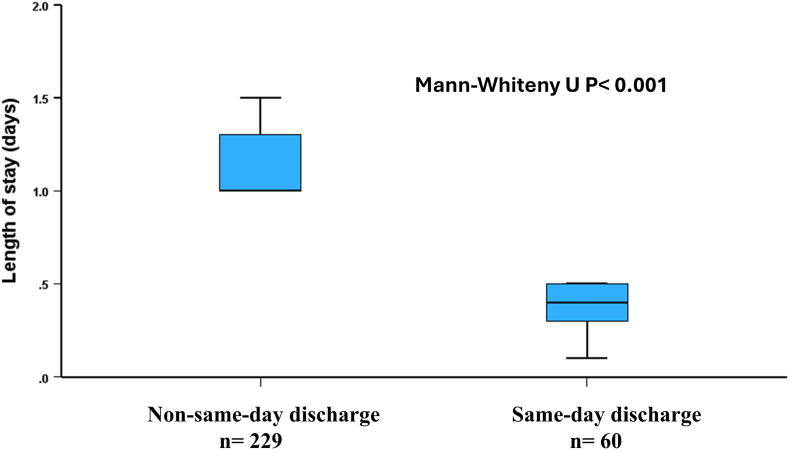
In-hospital length of stay in same-day and non–same-day discharge transcatheter aortic valve implantation.

The median length of stay was significantly lower in patients who underwent SDD TAVI than that in the group who had at least a single night in-hospital stay (9.5 [7-12] vs. 24 [24-31] hours, *p* < 0.001) (Figure 2). Patients who develop conduction abnormalities that did not necessitate pacemaker implantation had a median length of stay of 29 (24-34) hours.

**Figure 3:**
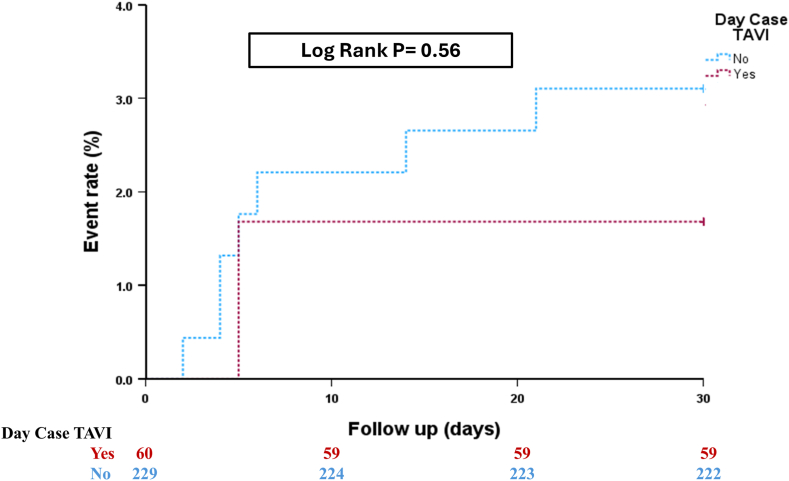
The primary endpoint in patients undergoing transcatheter aortic valve implantation (TAVI) stratified according to their same-day-discharge strategy.

The primary endpoint was reported in 2.8% of the whole cohort with no significant difference among patients who underwent SDD TAVI and those who did not (1.7% vs. 3.1%, hazard ratio 0.54, [95% CI 0.07-4.40], *p* = 0.56) (Figure 3, Table 2, Table 3). This relationship did not change even after adjustment for age, Society of Thoracic Surgeons score, obstructive lung disease, renal function, presence of pacemaker before TAVI, degree of LV function, and the presence or new onset of LBBB [hazard ratio 0.66, (95% CI 0.07-5.80), *p* = 0.71]. There was only one death and one late vascular complication in the non-SDD TAVI group. There were 6 (2.1%) patients who had late PPM following hospital discharge, and out of these, 5 patients underwent non-SDD TAVI.

**Table 2:** Procedural, electrical, and echocardiographic outcomes of included patients

Outcome	Whole cohort (*n* = 289)	Non–same-day discharge TAVI (*n* = 229)	Same-day discharge TAVI (*n* = 60)	*p* Value
Self-expanding valve	166 (57%)	135 (59%)	31 (52%)	0.31
Evolut	114 (69%)	94 (70%)	20 (65%)	
Navitor	40 (24%)	34 (25%)	6 (19%)	
Others	12 (7%)	7 (5%)	5 (16%)	
Valve size	26 [26-29]	26 [26-29]	26 [26-29]	0.81
Valve-in-valve	2 (0.7%)	1 (0.4%)	1 (1.7%)	0.27
Predilation	109 (38%)	90 (39%)	19 (32%)	0.28
New/existing LBBB	115 (40%)	101 (44%)	14 (23%)	0.03
Change in PR interval, milliseconds (mean ± SD)	15 ± 35	15 ± 26	13 ± 57	0.68
Change in QRS duration, milliseconds	11 [2-40]	12 [0-48]	9 [4-19]	0.19
Mean gradient, mmHg	10 ± 5	10 ± 5	10 ± 5	0.75
Peak gradient, mmHg	19 ± 8	19 ± 8	19 ± 8	0.91
Effective orifice aortic valve area, cm^2^	1.7 ± 0.5	1.7 ± 0.5	1.7 ± 0.5	0.94
Mean gradient >20 mmHg	10 (4%)	6 (3%)	4 (7%)	0.13
Preserved LV function, (EF > 50%)	191 (83%)	143 (81%)	48 (89%)	0.19
Severe PVL	1 (0.3%)	0 (0%)	1 (1.7%)	0.21

*Note.* Transthoracic echocardiography parameters are reported at 6 wk after the TAVI procedure. Data presented as mean ± standard deviation, median [interquartile range], or n (%), where applicable.

Abbreviations: EF, ejection fraction; IQR, interquartile range; LBBB, left bundle branch block; LV, left ventricular; PVL, paravalvular regurgitation; SD, standard deviation; TAVI, transcatheter aortic valve implantation.

**Table 3:** Clinical outcomes of included patients at 30 d

Outcome	Whole cohort (*n* = 289)	Non–same-day discharge TAVI (*n* = 229)	Same-day discharge TAVI (*n* = 60)	*p* Value
Primary endpoint	8 (2.8%)	7 (3.1%)	1 (1.7%)	0.56
Death	1 (0.3%)	1 (0.4%)	0 (0%)	0.61
Vascular complications	1 (0.3%)	1 (0.4%)	0 (0%)	0.61
Neurological events	2 (0.7%)	2 (0.9%)	0 (0%)	0.63
New pacemaker implants	6 (2.1%)	5 (2.2%)	1 (1.7%)	0.64

Data presented as n (%). Abbreviation: TAVI, transcatheter aortic valve implantation.

Patients who underwent self-expanding valve placement had a comparable primary endpoint to those who underwent balloon-expandable valve placement (3% vs. 2.4%, respectively, *p* = 0.51) in the whole cohort and irrespective of whether they were discharged on the same day or not. In addition, none of the TAVI devices was associated with the primary endpoint on the multivariate analysis. The number of patients who were readmitted for a PPM was also similar between the 2 platforms (1.8% vs. 2.4%, *p* = 0.51).

There was one case of hospital readmission in patients who underwent SDD TAVI. This was due to low-grade temperature (subclinical rise in body temperature). The patient was kept in hospital for monitoring and was discharged with no change in their active clinical care.

## Discussion

The current study reports the clinical outcomes of patients undergoing the SDD TAVI procedure. The main findings from this study can be summarized as follows: First, the SDD TAVI procedure appears safe and feasible for a selected patients undergoing an elective transfemoral procedure, although the total number of events was relatively low; second, the rate of late PPM was also low, and criteria that allowed same-day-discharge TAVI were not associated with increased risk of pacemaker at 30 days; finally, the use of a self-expanding valve was not linked to a higher events rate or late pacemaker implantation if patients met the predefined protocol criteria for SDD TAVI.

Advances in technology coupled with increased institutional and operator experience as well as better understanding of procedural risks have streamlined TAVI procedures and allowed direct comparison of clinical outcomes in low-risk patients undergoing surgical AVR.^3^^,^^4^ Importantly, TAVI has consistently resulted in shorter length of stay and early hospital discharge compared to surgical AVR.^5^^,^^6^ The safety of early discharge is a well-established approach in contemporary TAVI.^16^^,^^17^ The FAST TAVI (Feasibility and Safety of Early Discharge After Transfemoral Transcatheter Aortic Valve Implantation) study reported a low event rate in patients who were appropriately discharged early from the hospital if they met strict criteria.^9^ Of note, the median length of stay was 2 days, and patients were included if they only received a balloon-expandable valve with a SAPIEN 3 transcatheter heart valve (Edwards Lifesciences).^9^ The Vancouver 3M (Multidisciplinary, Multimodality, but Minimalist) reported excellent safety outcomes in 327 patients (out of 411 screened patients) who received a balloon-expandable valve with the SAPIEN 3 platform.^8^ The authors highlighted the feasibility of next-day discharge, irrespective of the center TAVI volume, supporting the generalizability of their findings.^8^

More recently, SDD following TAVI was proposed to maintain procedural volume during COVID-19 pandemic.^18^ The Multicentre PROTECT TAVR study reported the safety and feasibility of SDD following TAVI in highly selected patients who are at low risk of adverse clinical events.^10^ Out of 2100 patients who underwent elective transfemoral TAVI, the authors only included 124 patients (5.9%) as candidates for SDD. Importantly, almost one-third of these patients had a pre-existing pacemaker, and they predominately received a balloon-expandable valve (97%).^10^ This was also borne out in a very small single center study that reported clinical outcomes in 13 patients following almost 2 years of screening.^19^

Collectively, previous studies highlighted the safety of early or SDD in highly selected patients. The presence of an existing pacemaker was relatively high in those studies. In contrast, our study included patients in whom a pacemaker was present in less than 1 in 7 patients. The risk of conduction block after TAVI is often the driver for extended hospital stay. Importantly, in our cohort who were discharged on the same day, patients with conduction abnormalities such as LBBB or RBBB were considered for SDD TAVI, if they met our protocol inclusion criteria. It is widely accepted that pre-existing RBBB is a strong predictor of high-degree atrioventricular block following TAVI.^20^, ^21^, ^22^ Importantly, this risk can persist up to 7 days, challenging the notion of keeping patients in hospital for 24-48 hours.^20^^,^^22^ Moreover, it remains to be determined whether this risk is modifiable by pre-emptively implanting a pacemaker and whether the risk/benefits ratio would be acceptable, particularly in low-risk patients. A recent study highlighted that almost one-third of patients who received a pacemaker with pre-existing RBBB required less than 1% ventricular pacing.^23^ Nonetheless, a prophylactic pacemaker in patients with pre-existing RBBB may be a reasonable approach in elderly patients. The role or pre-existing LBBB on high-degree AV block in patients undergoing TAVI has also been assessed.^24^, ^25^, ^26^ There is conflicting data on the association of LBBB and early pacemaker implantation following the TAVI procedure. Fischer et al.^24^ reported 50% increased risk of early pacemakers within 30 days, but this risk was not translated into an increase in mortality. Interestingly, this risk was not evident after 30 days, and whether pacemaker decision in this group is based on perceived, rather than actual, risk may need to be considered. A more recent study reported the lack of association between pre-existing LBBB and early pacemaker implantation.^25^ Among 5996 patients who underwent transcatheter aortic valve replacement, 4.6% had pre-existing LBBB, and their rate of new pacemaker was 2.9% compared to 3.1% in patients without LBBB.^25^ Overall, this supports our approach in allowing SDD in patients without significant ECG changes, despite pre-existing LBBB.

Previous studies have also focused on the safety of early discharge using balloon-expandable platform. Data on same-day or next-day discharge for self-expanding valve placement remain limited. Ordoñez et al.^27^ reported the safety outcomes of next-day discharge using an ACURATE neo/neo2 valve (Boston Scientific, Marlborough, Massachusetts). More recently, a meta-analysis of 6 studies including 3519 patients reported favorable outcomes of patients who had SDD.^28^ The pooled sample size of patients who underwent SDD in this meta-analysis was 318, of whom a self-expanding valve was implanted in only 27 (8.4%) patients. In contrast, our single-center study reported the safety outcomes of 31 patients with a self-expanding valve who were discharged on the same day following the TAVI procedure. This highlights the current limitation of existing literature regarding the feasibility of SDD for this group and illustrates the significance of our study findings in demonstrating the safety of SDD for patients undergoing self-expanding valve placement. Importantly, there was no difference in the primary endpoint of death, late vascular complications, bleeding, all strokes, or new PPMs between patients who received self-expanding and balloon-expandable valves. More specifically, the rate of unplanned late pacemaker placement was comparable between the two platforms and irrespective of the duration of in-hospital stay.

Our study has several limitations. This was a single-center study that included all elective patients who were scheduled for TAVI procedures. The results of the study cannot be extrapolated to patients who were transferred for urgent TAVI. The primary endpoint was reported at 30 days, and longer follow-up may be needed, particularly for those with pre-existing conduction abnormalities. Finally, the total number of events was relatively low (8 events), and type II statstical error cannot be excluded. Therefore, the lack of statistical difference in clinical outcomes among patients who did or did not undergo SDD should not be interpreted as equivalent, and studies with a larger sample size are required to confirm our findings. In addition, the imbalance between the 2 groups and the lack of propensity match analysis need to be taken into consideration when interpreting the results.

In conclusion, through the use of a robust protocol, we have shown that SDD TAVI is a safe and feasible approach in selected patients who underwent uneventful elective procedures. Same-day and early discharge following TAVI include better patient experience and more efficient utilization of healthcare resources. Future randomized studies are required to support these findings.

## Ethics Statement

The study was part of clinical audit and formal research ethics approval was not required. All patients provided written informed consent for their procedures, and the study was conducted according to the Declaration of Helsinki and Good Clinical Practice guidelines.

## Funding

The authors have no funding to report.

## Disclosure Statement

The authors report no conflict of interest.
