# Supplementary material for: Safety of Same-Day Discharge After Elective Transcatheter Aortic Valve Implantation With Balloon- and Self-Expanding Valves: A Prospective Single-Center UK Study
Source: Struct Heart. 2025 Sep 5;9(11):100728. doi: 10.1016/j.shj.2025.100728 (PMC12605078; doi:10.1016/j.shj.2025.100728)
Supplement: Supplementary table [file mmc1.docx]

**Supplementary Table: Protocol for same-day discharge trans-catheter aortic valve implantation**

| Pre-procedure | Planned transfemoral approach under local anaesthetic  No significant cognitive impairment or frailty (defined as Katz score above 4)  Social support, including the availability of an individual to stay with the patient for the first night |
| --- | --- |
| Intra-procedural | Uncomplicated procedure, including satisfactory vascular closure  Satisfactory technical success including less than moderate aortic regurgitation on angiography  No significant change in electrocardiogram immediately post procedure for patients without cardiac devices  Contrast volume less than three times estimated glomerular filtration rate |
| Post-procedure | Supine for 2 hours, sat up for 1 hour, and fully ambulatory for 4 hours  Post procedure observations are within normal parameters  Satisfactory access site with no significant bleeding or haematoma  Telemetry for 4 hours  No significant electrocardiogram changes immediately before discharge  No new onset neurological disturbance |
| Post-discharge | Telephone follow up call from TAVI Specialist Nurse within 24 hours  Transthoracic echocardiography at 6 weeks post-procedure |
